# Oligocene deep ocean oxygen isotope variations primarily driven by temperature

**DOI:** 10.1038/s41561-025-01878-y

**Published:** 2026-01-07

**Authors:** Flavia Boscolo-Galazzo, Victoria E. Taylor, Eirik V. Galaasen, Diederik Liebrand, Edward Gasson, Edoardo Dallanave, Alvaro Fernandez-Bremer, Jakub Witkowski, Steve M. Bohaty, A. Nele Meckler

**Affiliations:** 1 MARUM, Center for Marine Environmental Sciences, University of Bremen, Bremen, Germany; 2 Department of Earth Science and Bjerknes Centre for Climate Research, University of Bergen, Bergen, Norway; 3 Department of Earth and Environmental Sciences, University of Manchester, Manchester, UK; 4 Earth Sciences Department, University College London, London, UK; 5 Department of Earth and Environmental Sciences, University of Exeter, Cornwall Campus, Exeter, UK; 6 Department of Earth Sciences, University of Milan, Milan, Italy; 7 Instituto Andaluz de Ciencias de la Tierra (IACT-CSIC), Armilla, Spain; 8 Institute of Marine and Environmental Sciences, University of Szczecin, Szczecin, Poland; 9 Institute of Earth Sciences, Heidelberg University, Heidelberg, Germany

**Keywords:** Palaeoclimate, Palaeoceanography

## Abstract

Our understanding of the long-term behaviour of global climate and the Antarctic ice sheet relies heavily on the oxygen isotopic composition of marine calcite (δ^18^O_calcite_), which reflects a combination of ocean temperature and the amount of water stored in ice sheets. On the basis of δ^18^O_calcite_, the Antarctic ice sheet has been interpreted as extremely dynamic in the Oligocene, 34–23 million years ago. Yet, the proposed continental-scale ice volume changes are challenging to reproduce with models and may be overestimated owing to a larger influence of temperature on the deep-sea δ^18^O_calcite_ than previously assumed. Here we present the first Oligocene record of orbital variability in deep ocean temperature based on benthic foraminiferal clumped isotope thermometry, a method affected only by temperature and independent of seawater chemistry. We find large, eccentricity-paced temperature variations of up to 4 °C, sufficient to explain the δ^18^O_calcite_ cycles without requiring continental-scale ice volume changes. This finding is consistent with the simulated stability of the Antarctic ice sheet, highlighting the importance of robust independent temperature reconstructions. Our results show that the temperature in the deep Southern Ocean, and possibly globally, is highly sensitive to the seasonal distribution of insolation in an Oligocene-like climate state warmer than today.

## Abstract (web summary)

Large benthic oxygen isotope fluctuations in the Oligocene Southern Ocean primarily represent deep water temperature changes, suggesting the Antarctic ice sheet volume was relatively stable, according to a clumped isotope record.

## Main

The geological past provides the opportunity to assess the behaviour of the climate system and the long-term stability of the Antarctic ice sheet under atmospheric carbon dioxide levels close to, and exceeding, those projected for the end of this century^1^. Both the global mean climate state and the behaviour of ice sheets are commonly inferred from deep ocean oxygen isotope records obtained from the microscopic calcite shells of benthic organisms called foraminifera (hereafter δ^18^O_calcite_)^2–4^. However, owing to a multitude of overlying influences on deep ocean δ^18^O_calcite_, isolating specific aspects of the climate system, such as continental ice volume or deep ocean temperature, requires additional assumptions or constraints (ref. ^5^ and references therein).

In the Oligocene (34–23 million years ago, Ma) when atmospheric CO_2_ ranged between 350 and 800 ppm^1^, the Arctic was ice-free^6^, whereas Antarctic-proximal sedimentological records indicate the presence of a continental-sized Antarctic ice sheet^7,8^. On the basis of the occurrence of large (~0.5–1.0‰) fluctuations in deep ocean δ^18^O_calcite_ records from the Atlantic and Pacific Oceans^9,10^, Antarctic ice volume in the Oligocene has been interpreted to have fluctuated by 34–90% of the modern ice sheet volume at orbital periodicities of 40 and 110 thousand years (kyr)^11–14^. Crucially, the upper range of these estimates would imply that, at elevated atmospheric CO_2_ relative to pre-industrial levels^1^, the Antarctic ice sheet could enter a dynamical state similar to the Northern Hemisphere ice sheets during the glacial–interglacial cycles of the Quaternary (the past 2.6 million years)^15^. Cyclical seaward advances and retreats of at least portions of the Antarctic ice sheet in the Oligocene are supported by ice-proximal sedimentological evidence^7,16^. However, ice sheet models are unable to reproduce the largest variations in ice volume interpreted from δ^18^O_calcite_ with a range of CO_2_ even larger than the current best estimates of Oligocene atmospheric CO_2_ (refs. ^1,17–19^). This is because strong stabilizing feedbacks limit ice sheet retreat from ice surface melting, and a topography without deep subglacial basins reduces the retreat of marine-based ice^17^. These conflicting lines of evidence currently obscure our understanding of the Antarctic ice sheet behaviour on orbital time scales.

A more stable Antarctic ice volume, as indicated by models, could be explained if the documented Oligocene variations in δ^18^O_calcite_ were driven primarily by factors other than continental ice volume. Benthic foraminiferal δ^18^O_calcite_ reflects both the temperature and the isotopic composition of seawater (δ^18^O_sw_), with the latter influenced globally by the volume and isotopic composition of continental ice, and to a lesser extent, regionally, by variations in surface ocean salinity at sites of deep-water formation^5,20^. Owing to the dominant influence of temperature, ice volume estimates from δ^18^O_calcite_ require either assumptions of relatively constant deep ocean temperatures^21,22^ or independent temperature reconstructions.

**Fig. 1:**
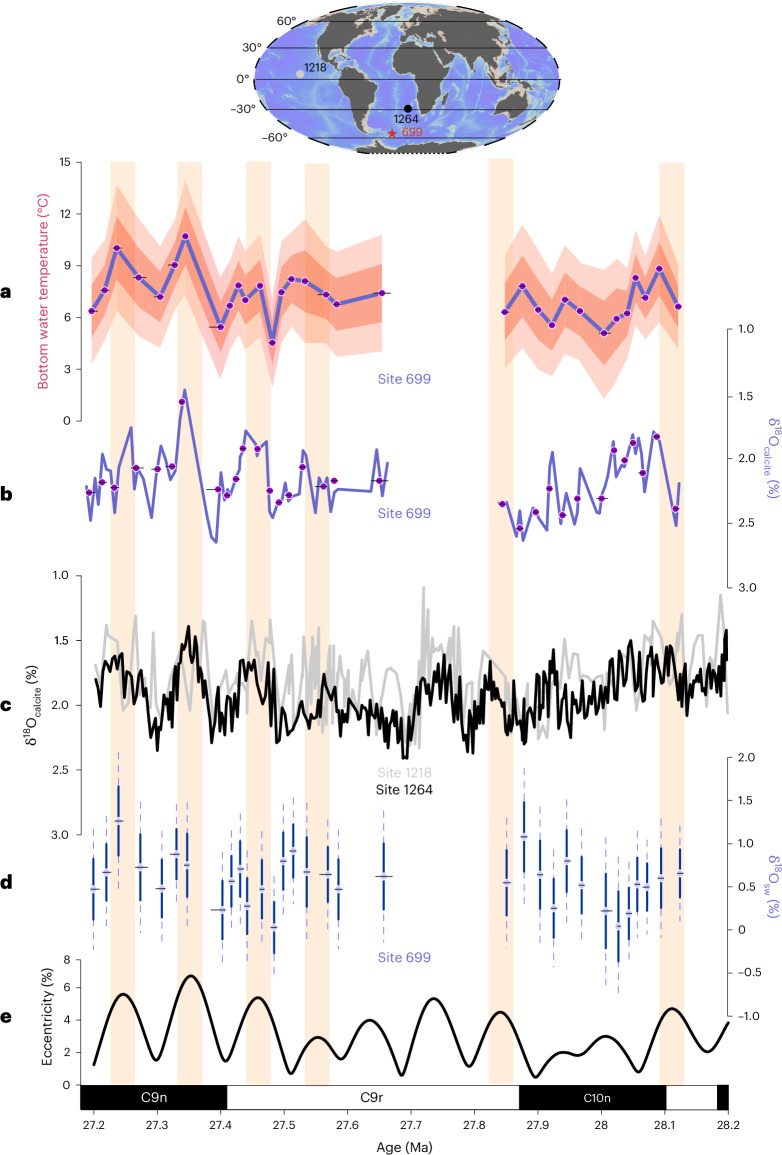
Eccentricity-paced cycles in bottom water temperature. **a**–**c**, Eccentricity-paced cycles in bottom water temperature (**a**), together with δ^18^O_calcite_ (‰VPDB) records from Site 699 (**b**), the Atlantic (Site 1264)^10^ and Pacific Ocean (Site 1218)^9^ (**c**). In **b**, δ^18^O_calcite_ is shown both for all samples (continuous purple line) and averaged in the same way as the Δ_47_ record (purple dots) to obtain the Site 699 record of δ^18^O_sw_ (‰VSMOW) (**d**). In **a** and **d**, the data are presented as mean values with light and dark envelopes in **a** and stippled and continuous error bars in **d** representing the 95% and 68% confidence intervals, respectively, derived from 20–46 measurements sourced from two to five individual sediment samples (Methods; Supplementary Table 2). **e**, The cycles in Earth’s orbital eccentricity^57^ with higher values indicating the times of greater southern hemisphere summer insolation during precession maxima. Black and white bars indicate the magnetic polarity stratigraphy at Site 699 (ref. ^58^). The yellow bands mark the 110-kyr eccentricity cycles captured at Site 699. The horizontal bars in **a**, **b** and **d** reflect the age ranges of the adjoining two to five samples combined for calculating the mean temperature and δ^18^O_sw_ values (Main and Methods). The inset map shows the modern location of ODP Sites 699, 1264 and 1218. Map generated with Ocean Data View^59^.

**Extended Data Fig. 1:**
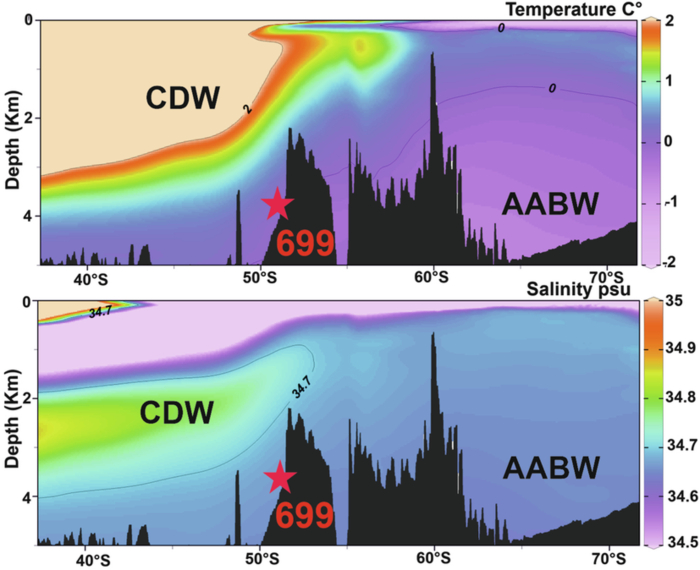
Present day bathymetry and water masses at Site 699. North-South cross-sections at 30°W showing the modern location of Site 699 with present-day temperature and salinity. Contours for temperature and salinity highlight the two main deep-water masses characterizing the area: CDW (Circumpolar Deep water) and AABW (Antarctic Bottom Water). Figure generated using https://odv.awi.de ref. ^59^.

**Extended Data Fig. 2:**
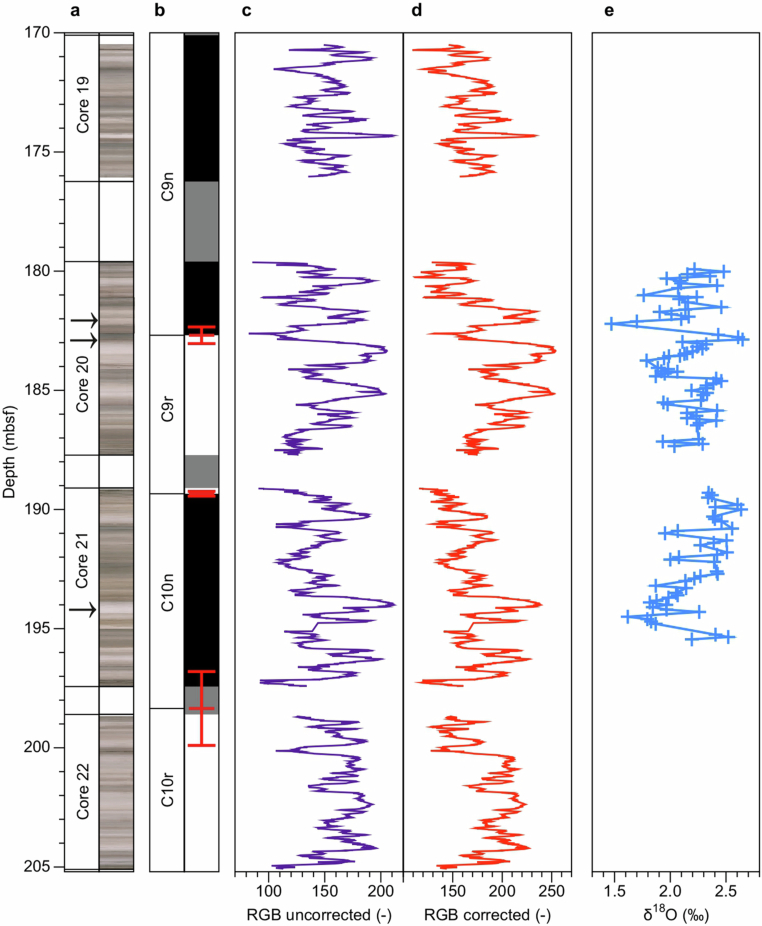
Site 699 stratigraphic data in the depth domain. (**a**) Hole 699 A Cores 19H to 21H were visually inspected for the presence of sedimentary cycles. Core color images show distinct cyclicity in the depth domain. (**b**) Magnetostratigraphy from Hole 699A^58^, where black (white) indicates a normal (reversed) magnetic polarity. Grey corresponds to coring gaps. Red error bars indicate uncertainty intervals of the exact magnetic reversal positions in the depth domain. Specifically, to each chron-reversal is assigned a mean-depth value indicated with a horizontal red bar. Mean-depth values are calculated based on the uppermost and lowermost magnetic chron positions, as reported by ref. ^58^, also used to calculate the error bars in the figure. The spread from the mean of the upper and lower depth uncertainties (error bars) ranges from ±0.1 to 1.5 meters. Note that the reversal from Chron C10r to C10n falls in the core gap in between Core 22H and Core 21H, and this reversal comes with a greater uncertainty. The study interval spans Cores 699A-20H and 21H, and Subchrons C9n to C10n. (**c**) Uncorrected red (R), green (G), blue (B), (RGB), data extracted from the core images (**a**). (**d**) RGB data corrected for uneven lighting conditions of core photographs. (**e**) Benthic foraminiferal δ^18^O data from Site 699. Arrows indicate the stratigraphic depths of foraminiferal samples used for SEM images shown in Extended Data Figs. 5–7.

**Extended Data Fig. 3:**
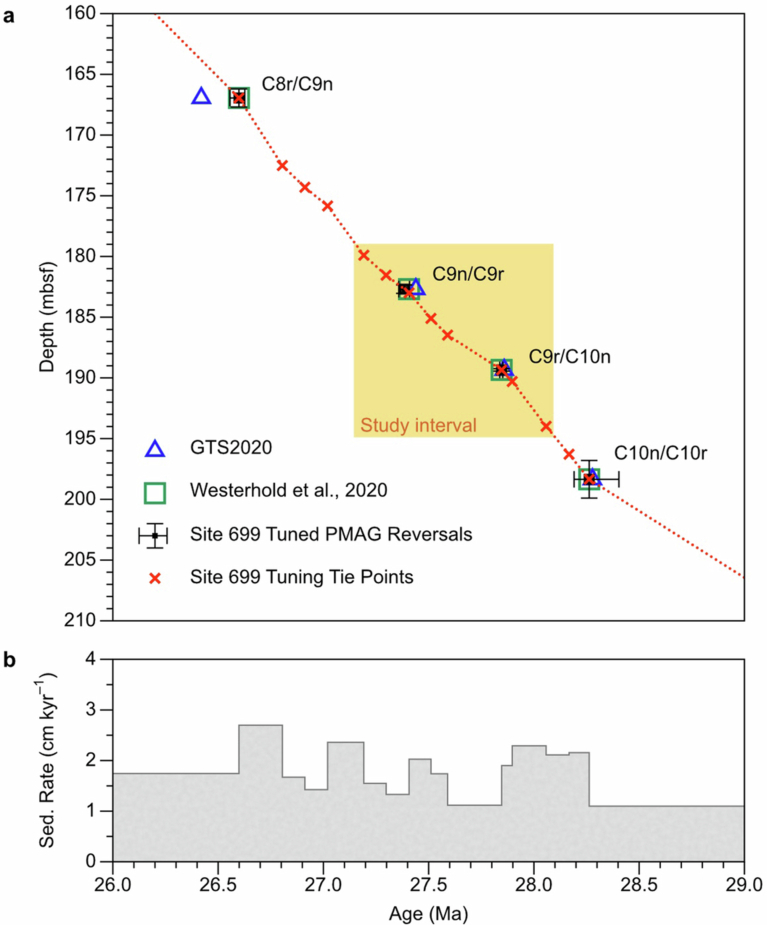
Site 699 initial magnetostratigraphic and final tuned age models. (**a**) Depth-age relationship. Our initial age model consisted of assigning the Westerhold et al.^3^ reversal ages to Site 699 magnetostratigraphic chron boundaries (ref. ^58^). Black error bars on the y-axis indicate the upper, mean, and lower depth position for each reversal boundary (see Extended Data Fig. 2 caption for an explanation). Black error bars on the x-axis indicate how the uncertainty in the depth-domain (uppermost, mean, and lowermost positions) translates into an age uncertainty. Note that upper and lower depth uncertainties with respect to the mean are equal (see y-axis), but that younger and older age uncertainties with respect to the mean vary from one another (see x-axis). This is resulting from changing depth-age relationships, that is, varying sedimentation rates, throughout our study interval. In the age domain the spread from the mean of the younger and older age uncertainties ranges from -0.009 and +0.005 Myr to -0.072 and +0.141 Myr. Subsequent alignment of the RGB record to the La2011_ecc3L^57^ eccentricity solution resulted in a more accurately constrained age control for Site 699. Our tuned reversal ages are within error in agreement with those of Westerhold et al.^3^, and apart from the C8r/C9n reversal, also with the GTS2020 ages^81^. (**b**) Site 699 sedimentation rates based on our eccentricity tuning. Sedimentation rates vary between ~1.1 and 2.7 cm kyr^−1^, values that are typical for a (hemi)pelagic deep marine site.

**Extended Data Fig. 4:**
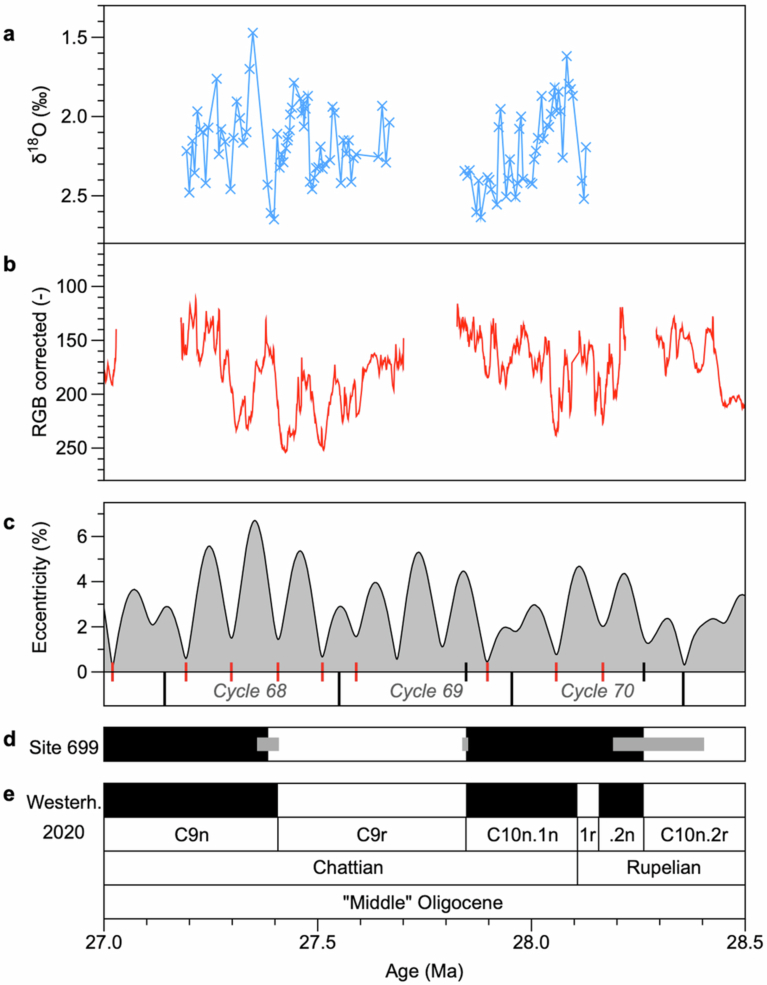
Site 699 astronomical age calibration. Astronomical age calibration was guided by Site 699 (**a**) benthic foraminiferal δ^18^O record, but mainly based upon (**b**) the RGB record from Hole 699A Cores 20H and 21H. Tuning target was (**c**) the La2011_ecc3L^57^ eccentricity solution. Tuning tie points based on an alignment of RGB (and δ^18^O) to eccentricity are indicated with red vertical lines. Black vertical lines are tie points based on the (**d**) Site 699 magnetic polarity reversals^58^, which were assigned the (**e**) Westerhold et al.^3^ ages. Within error, (**d**) and (**e**) agree with one another. Chron boundary names are indicated.

**Extended Data Fig. 5:**
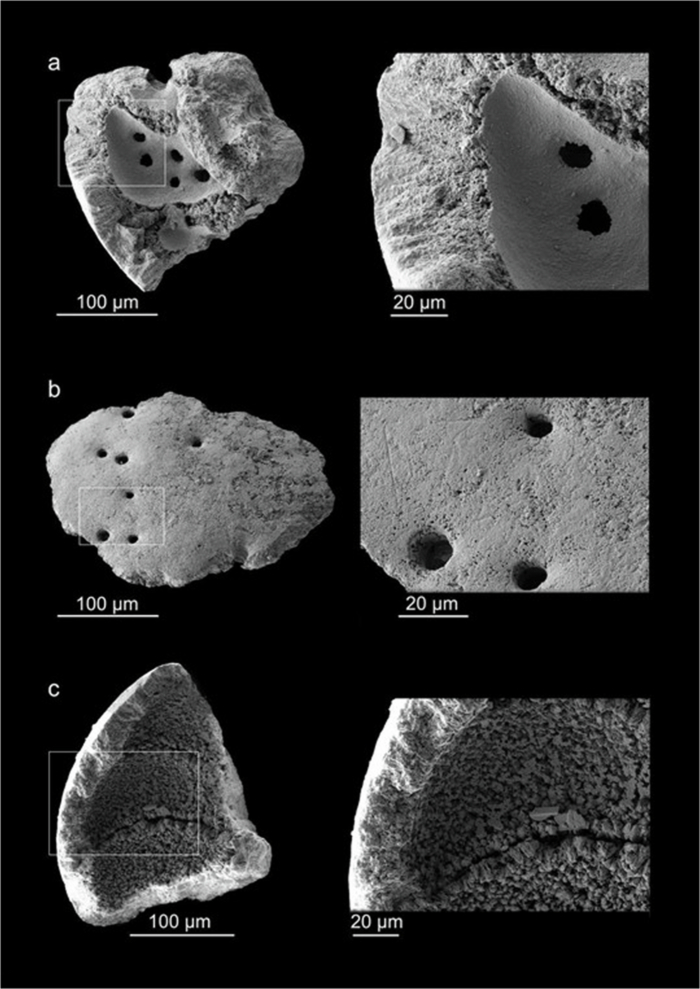
SEM images of cleaned fragments of *Cibicidoides* spp. Sample 699A-20H-2, 100-105 cm (182.10–182.13 mbsf). Panels **a**, **b**, and **c** show whole fragments and the images on the right are zoomed in (see panels **a**, **b**, **c** for locations).

**Extended Data Fig. 6:**
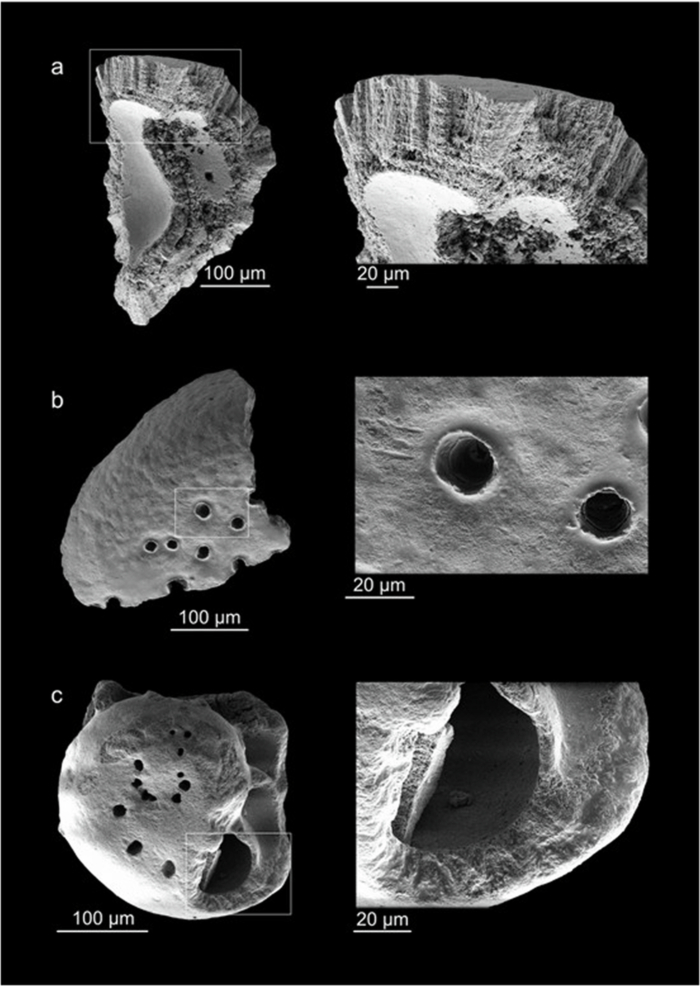
SEM images of cleaned fragments of *Cibicidoides* spp. Sample 699A-20H-3, 45-50 cm (183.05–183.08 mbsf). Panels **a**, **b**, and **c** show whole fragments and the images on the right are zoomed in (see panels **a**, **b**, **c** for locations).

**Extended Data Fig. 7:**
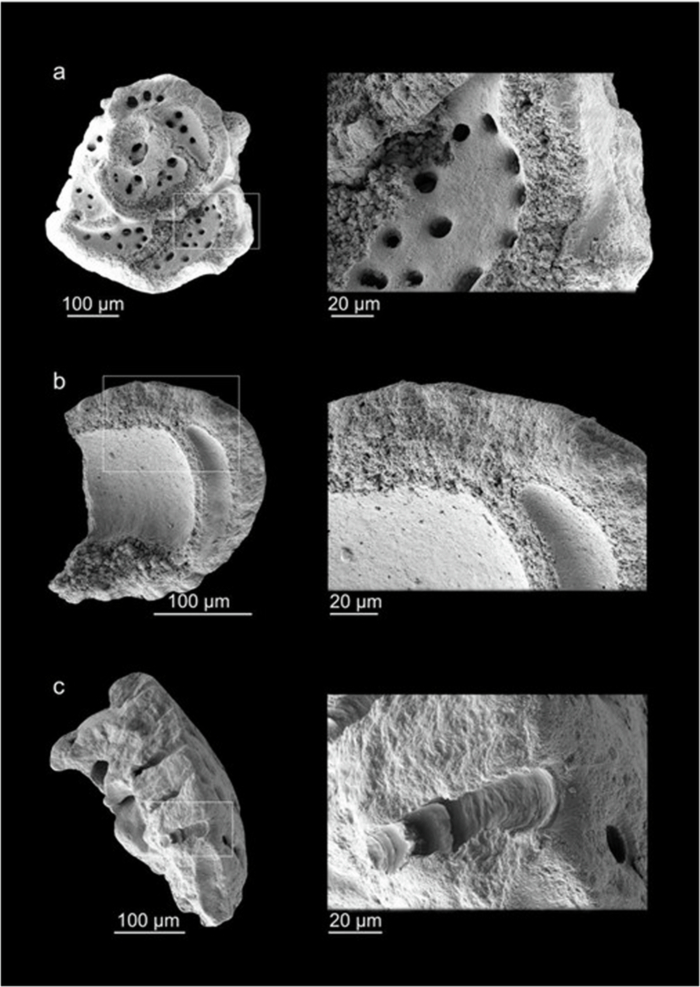
SEM images of cleaned fragments of *Cibicidoides* spp. Sample 699A-21H-4, 50-53 cm (194.10–194.13 mbsf). Panels **a**, **b**, and **c** show whole fragments and the images on the right are zoomed in (see panels **a**, **b**, **c** for locations).

**Extended Data Fig. 8:**
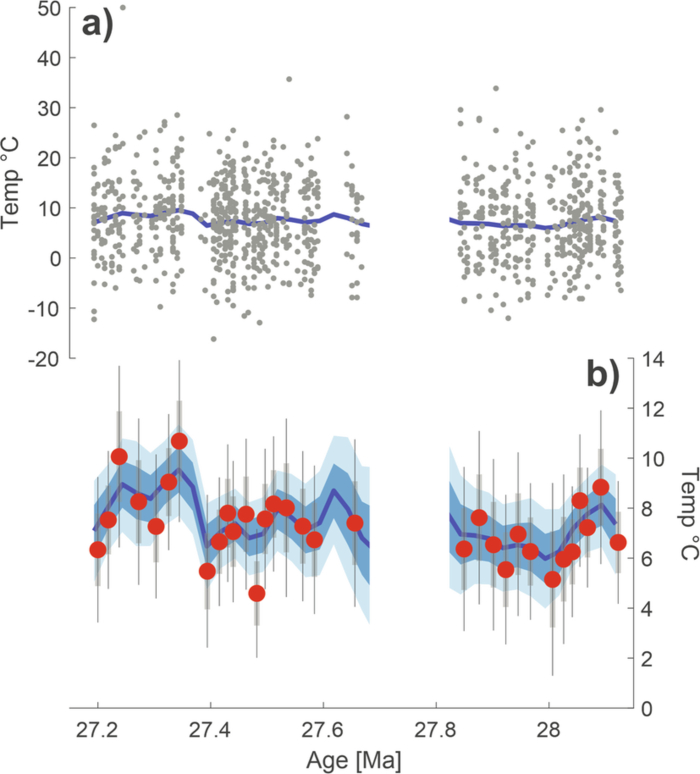
Gaussian Filter. (**a**) Replicate analyses (grey circles) and filter median values (blue line). (**b**) Filter median values (blue line) and 68% and 95% confidence intervals shown as dark blue and light blue envelopes. These were calculated with the simulation 16th – 84th and 2.5th – 97.5th percentiles, respectively. Red circles are the temperature estimates for the temperature groups expressed as mean values. Errors on temperature groups are the 68% and 95% confidence intervals derived from 20-46 measurements sourced from 2-5 individual sediment samples.

Here, we derive independent estimates of temperature using clumped isotope thermometry (Δ_47_)^23^ to test for orbital-scale deep ocean temperature variability during the interval spanning 28.2–27.2 Ma, which encompasses the largest δ^18^O_calcite_ cycles of the Oligocene^9,10^ (Fig. 1). Our aim is to test whether deep ocean temperatures vary on eccentricity timescales and, if so, to determine the proportion of the δ^18^O_calcite_ signal driven by temperature variability. Δ_47_ is independent of δ^18^O_sw_ and seawater elemental ratios, which affect the traditional trace element-based paleothermometers such as foraminiferal Mg/Ca ratios^23,24^, and it does not appear to be measurably affected by organism biology^23,25,26^. Because of their microscopic size, the application of Δ_47_ to foraminifera has until recently been limited by the requirement of large sample amounts (more than 10 mg)^27^. Advances in analytical techniques^28,29^, however, have increased the scope of possible applications of this proxy (for example, refs. ^30–32^). Here, we present the first deep ocean temperature record derived by clumped isotopes that resolves the high-amplitude 110-kyr δ^18^O_calcite_ cycles observed in the Oligocene. The 110-kyr cycles (that is, the combined 95-kyr and 125-kyr eccentricity components) reflect the shortest periodicity of changes in the shape of Earth’s orbit, which, in association with other orbital parameters, controls the amount and seasonal distribution of incoming solar radiation (Fig. 1). Our independent deep ocean temperature record comes from Ocean Drilling Program (ODP) Site 699 in the subpolar Southern Ocean at a present-day water depth of 3,705 m (ref. ^33^) (Fig. 1 and Extended Data Fig. 1). An orbital chronology was developed using sediment colour reflectance and δ^18^O_calcite_ records (Extended Data Figs. 2–4 and Supplementary Table 1). A total of 1,082 individual benthic foraminiferal Δ_47_ measurements from 99 discrete samples (Extended Data Figs. 5–8 and Supplementary Table 2) were binned into 32 temperature data points (Methods) that trace the large δ^18^O_calcite_ cycles characterizing the mid-Oligocene^9,10^ (Fig. 1), with an average temporal resolution of 28 kyr (Fig. 1). This binning is necessary because, with the small-aliquot approach applied here, the precision of individual Δ_47_ measurements is insufficient for paleoclimatic interpretation (Methods). The fidelity of our Δ_47_ binning is confirmed by good agreement with a moving window Gaussian filter, averaging temperature with a resolution of 25 kyr (Methods; Extended Data Fig. 8).

**Extended Data Fig. 9:**
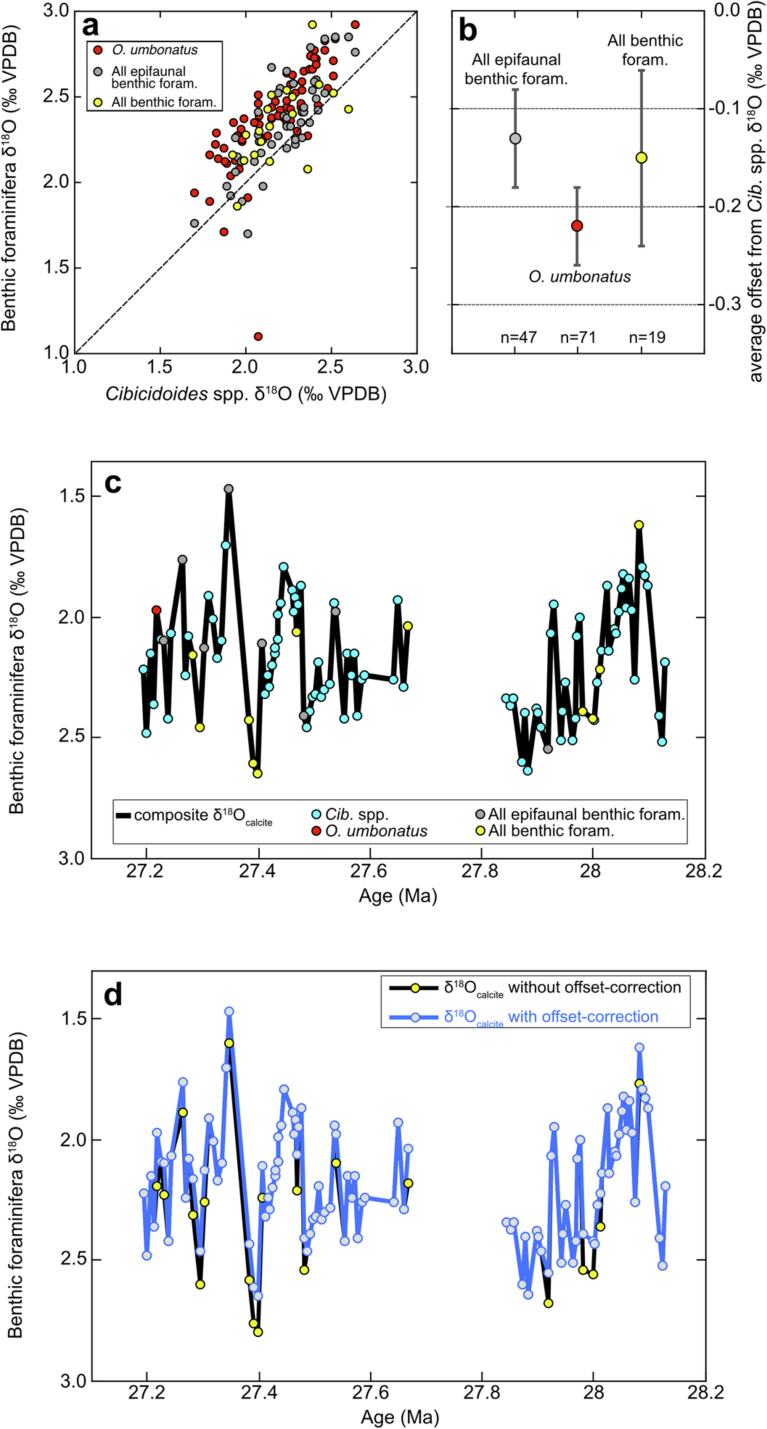
Constructing a species-corrected Site 699 benthic foraminifera δ^18^O_calcite_ record. (**a**) Cross-plot of δ^18^O_calcite_ from *Cibicidoides* spp. versus other key taxonomic groupings used here (red: *O. umbonatus*; grey: aliquots of mixed epifaunal benthic foraminifera species; yellow: mix of all benthic foraminifera present) from individual samples where measurements of both *Cibicidoides* spp. and other were possible. (**b**) The averaged δ^18^O_calcite_ offset from *Cibicidoides* spp. of the groupings in (**a**) for all samples containing dual measurements, with 95% confidence intervals (vertical bars) and number of samples containing measurements of a specific grouping as well as *Cibicidoides* spp. displayed at the bottom. (**c**) The composite Site 699 benthic foraminifera δ^18^O_calcite_ record (black line) plotted versus age, with taxonomic groups contributing to the record shown as coloured circles (blue: *Cibicidoides* spp.; red: *O. umbonatus*; grey: all epifaunal benthic foraminifera; yellow: all benthic foraminifera), constructed by correcting for the averaged δ^18^O_calcite_ offsets (see (**b**)) of the other groupings to that of *Cibicidoides* spp. (**d**) Comparison of the composite benthic foraminifera δ^18^O_calcite_ record with the offset-correction applied (blue line, grey circles) and without an offset-correction (black line, yellow circles).

The δ^18^O_calcite_ data obtained for each sample as a co-product of the Δ_47_ measurements (Methods) provide a higher-resolution record that reproduces the large eccentricity-paced δ^18^O_calcite_ cycles observed at other sites^9,10^ (Fig. 1, Extended Data Fig. 9 and Supplementary Table 2). The δ^18^O_calcite_ data were additionally binned in the same way as the Δ_47_ data and combined with their corresponding temperature data points, to extract the residual variability in δ^18^O_sw_ (Methods; Fig. 1).

## Warm mid-Oligocene bottom waters

Mid-Oligocene bottom water temperatures at Site 699 varied between 4.3 °C (±1.8 °C) and 10.8 °C (±1.5 °C) (uncertainty reported at 68% confidence intervals; Methods) within the study interval and averaged 7.2 °C ± 1.4 °C (Fig. 1), substantially warmer than the present-day bottom water temperature of 0 °C at this site^34^ (Extended Data Fig. 1). When compared with reconstructions based on δ^18^O_calcite_ for the study interval, our Δ_47_-derived mean deep ocean temperature is significantly warmer than the 1.4 °C estimate of Rohling et al.^13^ but within error of the 5.6 °C estimate of Cramer et al.^35^. Our reconstructed temperature is close to the mean deep ocean temperature of 6.5–7 °C obtained from Mg/Ca thermometry for the interval 28–26 Ma (ref. ^35^), although the absolute temperatures derived from Mg/Ca are highly dependent on calibration choices (for example, refs. ^14,36^), and also match with clumped isotope-based estimates for the mean Oligocene temperature in the deep North Atlantic (7.7 °C ± 1.1 °C between 33.5 and 24.4 Ma)^37^. The overall deep ocean warmth reconstructed for the mid-Oligocene furthermore corresponds well with the reconstructed contemporaneous sea surface temperatures seasonally exceeding 10 °C at sites in the Southern Ocean^38^ and on the Antarctic margin^39–41^, the likely source region for the bottom water bathing Site 699 (refs. ^42,43^).

The calculated δ^18^O_sw_ values average 0.57‰ ± 0.34‰ within the study interval. If reflecting only continental ice volume, this value would indicate a substantially larger ice volume than at present. The direct translation of δ^18^O_sw_ into continental ice volume (for example, ref. ^12^), however, is complicated by the additional influences on δ^18^O_sw_, including δ^18^O_ice_ of the ice sheet and regional, salinity-related changes in δ^18^O_sw_. Large interbasin salinity gradients have been reconstructed for the deep ocean in the mid-Pliocene^44^, highlighting that this factor must be considered when interpreting δ^18^O_sw_, especially in climate states warmer than today. We note that both the absolute temperature and δ^18^O_sw_ would be lower by around 1.5 °C and 0.35‰ respectively, with alternative clumped isotope calibrations^45,46^ (Methods). This difference in absolute temperatures, however, does not exceed uncertainty (Methods), and, crucially, the relative changes in temperature and their relationship with observed variations in δ^18^O_calcite_ remain independent of our calibration choice.

## Eccentricity-paced variability in bottom water temperature

**Fig. 2:**
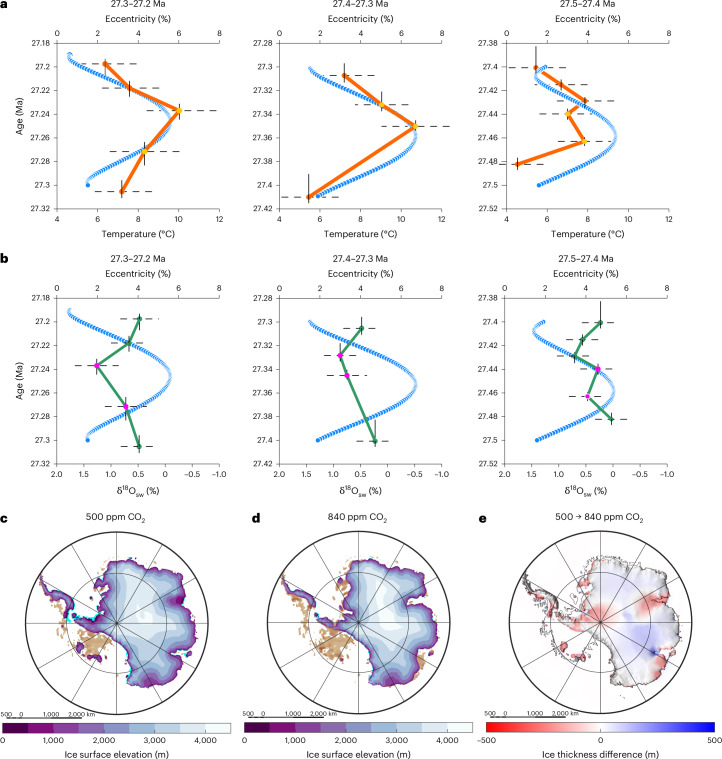
An early Antarctic ice sheet resilient to climate change. **a**,**b**, The largest 110-kyr cycles in the younger part of our record from Site 699, for which the age model is best constrained, showing eccentricity maxima matching maxima in bottom water temperatures (**a**) and the absence of consistent cyclical changes in δ^18^O_sw_ (‰VSMOW) (**b**), suggesting no orbital variability in Antarctic ice volume. **c**–**e**, The results in **a** and **b** are consistent with Oligocene ice sheet model simulations^17^ showing minimal ice volume variability between 500 ppm (**c**) and 840 ppm (**d**) atmospheric CO_2_ due to melting in coastal regions being compensated by increased ice thickness inland at higher CO_2_ (**e**). The yellow and purple dots in **a** and **b** indicate values included in the eccentricity maxima groups for the purpose of testing statistical significance of peak versus background values (Methods; Extended Data Fig. 10). In **a** and **b**, the data are presented as mean values with horizontal stippled lines representing the 68% confidence intervals of the proxy data derived from 20–46 measurements sourced from two to five individual sediment samples (Methods; Supplementary Table 2). The vertical lines reflect the age ranges of the adjoining two to five samples combined for calculating the mean temperature and δ^18^O_sw_ values (Main and Methods). The eccentricity values are from ref. ^57^. Panels **c**–**e** adapted from ref. ^17^ under a Creative Commons license CC BY 4.0.

At Site 699, sediment properties (Methods), δ^18^O_calcite_ and bottom water temperature show a cyclicity with a dominant 110-kyr (short eccentricity) pacing throughout the studied interval (28.2 to 27.2 Ma; Fig. 1 and Extended Data Figs. 2 and 4). The studied interval captures a total of six 110-kyr cycles (Fig. 1 and Extended Data Fig. 9), with the youngest three being the largest 110-kyr cycles of the Oligocene and corresponding to up to 1‰ changes in δ^18^O_calcite_, globally^9,10^ (Fig. 1). In the South Atlantic, these δ^18^O_calcite_ cycles have been interpreted to reflect changes in Antarctic ice volume equivalent to 100% of the modern East Antarctic ice sheet^11^. By contrast, our record shows distinct temperature changes of ±2.8–4.4 °C (±1.4 °C) associated with each of the largest δ^18^O_calcite_ cycles (Δδ^18^O_calcite_ ± 0.6–1‰) (Fig. 1). This amplitude of eccentricity (110-kyr)-paced temperature variations in the mid-Oligocene is slightly larger than 2–3 °C obliquity (40-kyr)-paced temperature variations reconstructed with Mg/Ca thermometry for the late Oligocene deep North Atlantic Ocean^12^. With an empirical relationship between δ^18^O_calcite_ and temperature of approximately −0.22‰ per degree Celsius^20,47^, our reconstructed changes in deep ocean temperature alone are in principle sufficient to explain the δ^18^O_calcite_ cycles observed at our site, without requiring continental-scale changes in Antarctic ice volume (Fig. 1). Our data clearly demonstrate that deep ocean temperatures can exhibit a high degree of variability on orbital timescales (Fig. 2), and therefore, δ^18^O_calcite_ cannot be interpreted to reflect δ^18^O_sw_ (and ice volume) changes in the absence of independent constraints on temperature. This suggests that the previous assumptions that led to the interpretation of continental-scale ice volume fluctuations based on Oligocene δ^18^O_calcite_ alone need revising^11^.

**Extended Data Fig. 10:**
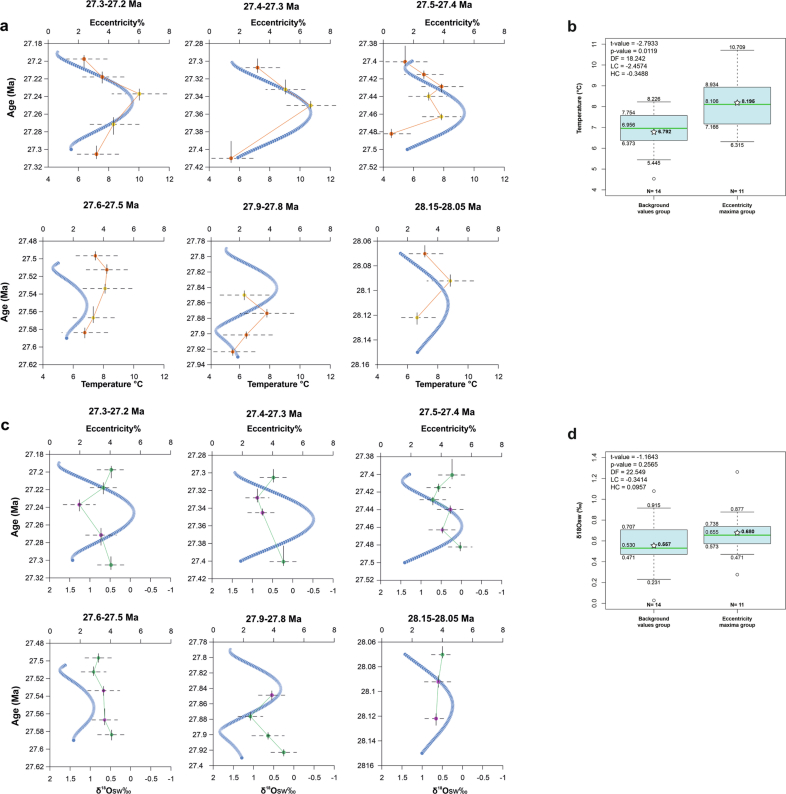
Statistical significance of temperature and δ^18^O_sw_ variability. (**a**) Δ_47_ temperatures plotted against the La2011_ecc3L Eccentricity Index from^57^ for the six 110-kyr eccentricity cycles captured here. Yellow dots indicate temperatures included in the eccentricity maxima group for the purpose of testing statistical significance of peak vs background values. Temperature data are presented as mean values with horizontal stippled lines representing the 68% confidence intervals of the proxy data derived from 20-46 measurements sourced from 2-5 individual sediment samples. Vertical lines reflect the age ranges of the adjoining 2-5 samples combined for calculating average Δ_47_ values. (**b**) Result of the Welch’s t-test^82^, a variation of the standard t-test that is more suitable for distributions with unequal variance, comparing the background temperature values with the ones at the eccentricity maxima. Each box-plot shows the mean (white star), median (green line), 25-75% range (boxes) and full range of values (whiskers) of the two groups. The numerical result of the Welch’s t-test is shown in upper-left-hand part of the figure, whereby DF= degree of freedom; LC and HC = 95% lower and higher (respectively) confidence boundaries of the estimated likely true difference between the two groups. (**c**) Same as (**a**) but for δ^18^O_sw_ data. Purple dots indicate values included in the eccentricity maxima group for the purpose of testing statistical significance of peak vs background values. (**d**) Same as (**b**) but for δ^18^O_sw_ data.

The large analytical uncertainty of our residual δ^18^O_sw_ data prevents detailed interpretations of this signal, leaving open the possibility of some ice volume-related changes in δ^18^O_sw._ Nonetheless, we do not see any evidence for systematic, large-scale changes in δ^18^O_sw_ on orbital timescales, as it would be expected if cyclical, continental-scale, waxing and waning of the Antarctic ice sheet was the primary driver behind the δ^18^O_calcite_ record (Methods; Fig. 2 and Extended Data Fig. 10). Given the large deep ocean temperature changes we observe, the δ^18^O_calcite_ cycles could only accommodate for continental-scale fluctuations in ice volume—equivalent to melting the modern East Antarctic ice sheet, resulting in ~59 m of sea-level change^14^—if large changes in salinity and/or in δ^18^O_ice_ were counteracting the ice volume component in the δ^18^O_sw_ signal (Fig. 1). The δ^18^O_ice_ varies with ice sheet size owing to the effects of changing altitude and transport distance, but these effects are of the opposing sign, and they would probably be insufficient to offset large-scale ice volume changes^48^. The scale of salinity-related change in δ^18^O_sw_ in the study area required to offset continental-scale ice volume changes would have to approach the difference observed between deep Atlantic and Pacific water masses in the Pliocene^44^. Further, any regional salinity-related change in δ^18^O_sw_ would need to be compensated for by opposing signals elsewhere in the ocean to maintain mass balance, which may be hard to achieve given the volume of deep water that was probably formed in the Southern Ocean. For these reasons, it seems unlikely that bottom water salinity at Site 699 changed to the extent required to fully mask the large ice volume-related changes proposed in earlier studies. Hence, the most parsimonious explanation to account for the mid-Oligocene eccentricity cycles captured in the deep ocean oxygen isotope records is that they were primarily driven by temperature, with possible small variations in ice volume not exceeding the lower end estimates from Oligocene sea-level change reconstructions^13,14^.

The warmest deep ocean temperatures at our site coincide with eccentricity maxima, within age model uncertainty (Methods; Figs. 1 and 2 and Extended Data Fig. 10). The magnitude of the temperature and δ^18^O_calcite_ changes appears to be proportional to the degree of change in eccentricity, with intervals of less pronounced eccentricity, such as between 28.1 and 27.9 Ma, corresponding to more muted temperature and δ^18^O_calcite_ variability (Fig. 1). Hence, our new temperature reconstructions suggest a strong response of deep ocean temperature in the Oligocene to orbital variations in insolation (Fig. 2).

The deep ocean temperature reflects the surface temperature in areas of deep-water formation^27^, which, for Site 699, and much of the global ocean during the Oligocene, were most probably located in the Southern Ocean^42,43^. Our record could thus reflect regional eccentricity-paced temperature variations in the surface ocean surrounding Antarctica, with implications for ocean overturning and associated heat and carbon sequestration in the deep ocean. Alternatively, the eccentricity-paced deep ocean temperature variations could reflect switches between two different water masses bathing Site 699, characterized by different temperatures, akin to the Circumpolar Deep Water and Antarctic Bottom Water today (Extended Data Fig. 1). Similar temperature reconstructions from other sites are needed to distinguish between these scenarios. Regardless, given its location in the deep Southern Ocean, Site 699 is probably representative for a substantial portion of the global deep ocean, indicating a strong eccentricity imprint on ocean circulation and heat distribution during the middle Oligocene.

## A resilient early Antarctic ice sheet

If the 110-kyr eccentricity-paced δ^18^O_calcite_ cycles observed in the deep ocean are dominated globally by temperature changes on the order of ~3–4 °C (Fig. 1), this may offer a way to reconcile ice sheet modelling, suggesting a relatively stable Oligocene Antarctic ice volume, with the deep ocean δ^18^O_calcite_ records. In the Oligocene, the Antarctic ice sheet was probably not marine-based as it is today due to elevated bedrock topography^17,49^ (Fig. 2). The warm surface ocean temperatures proximal to Antarctica^39–41^ would have furthermore prevented an extensive marine-based ice sheet from forming. Models suggest that a land-based ice sheet is much less susceptible to large-scale melting compared with marine-based ice sheets, even under high CO_2_ concentrations (>1,000 ppm), resulting from self-stabilizing feedbacks caused by surface-elevation mass balance and albedo^17,19,49–55^. In model simulations that incorporate data-constrained Oligocene bedrock topographies for Antarctica, the ice volume loss obtained for a CO_2_ change from 500 to 840 ppm, in line with Oligocene estimates^1^, is negligible^17^ (<0.1%) (Fig. 2). For simulations with a low concentration of atmospheric CO_2_ of 280 ppm, which is lower than proxy reconstructions^1^, the ice volume change is equivalent to 25% of the modern-day ice volume^17,19^. In these simulations, melting in Antarctic coastal areas is counteracted by the increased ice thickness in the interior due to more abundant snowfalls in a warmer climate (Fig. 2). Therefore, changes in the spatial extent of the Antarctic ice sheet^7,16^ may not necessarily correspond to large ice volume changes (Fig. 2) and can explain changes in deep ocean temperatures decoupled from Antarctic ice volume^41,56^. Our work thus supports a very different response of the middle Oligocene Antarctic ice sheet to orbital variations compared with the ice sheets of the Quaternary at substantially lower levels of atmospheric CO_2_.

Our new data show temperature fluctuations in the deep ocean of up to 4 °C consistent with a mostly terrestrial Oligocene Antarctic ice sheet between 28 and 27 Ma that may have responded to orbital forcing by changes in its spatial extent but with relatively small variations in ice volume. This finding contradicts previous interpretations of δ^18^O_calcite_ fluctuations being the expression of continental-scale glacial–interglacial cycles in Antarctic ice volume. The large, eccentricity-paced (110 kyr) deep water temperature swings we reconstruct for the Southern Ocean (Figs. 1 and 2) suggest a climate state that is highly sensitive to external forcing during the middle Oligocene. At this time, the ocean, rather than ice sheet volume, was the primary component of the climate system mediating and responding to orbital variability in insolation. This may be an inherent feature of a warmer-than-present-day climate state such as the Oligocene. Our findings stress the importance of robust deep ocean temperature reconstructions and the need for more such records to reconcile our understanding of the Antarctic ice sheet from both the geological record and model simulations, and to understand the behaviour of the climate system in an Oligocene-like state characterized by the unipolar glaciation on Antarctica.

## Methods

### Study site

ODP Site 699 (51°32.537′ S, 30°40.619′ W) was drilled as a single hole (Hole 699A) in the Atlantic sector of the Southern Ocean at a water depth of 3,705 m, underneath the Antarctic Circumpolar Current (ACC) and Circumpolar Deep Water, and is today bathed by Antarctic Bottom Water^33,60^ (Extended Data Fig. 1). The paleolatitude of Site 699 in the Oligocene was very close to the modern^61^. The high abundance of siliceous microfossils in studied cores 699A-20H and 21H indicate a high productivity regime typical of the ACC region already in the Oligocene. This is corroborated by micropaleontological evidence^62^ indicating that, since the early Oligocene, the Atlantic Ocean south of 50 °S was under the influence of the ACC and had a polar oceanographic regime.

### Age model and orbital tuning

Sediments recovered at Site 699 possess a clear characteristic remanent magnetization, determined by means of shipboard continuous measurements on the archive halves and integrated by onshore analysis of discrete samples^58^. The newly generated diatom biostratigraphy supports the magnetic polarity correlation of cores 699A-20H and 699A-21H within subchrons C9n–C10n. This assignment is based on the occurrence of the following marker taxa: *Rocella vigilans*, *Kozloviella minor* and *Cestodiscus trochus*. Especially *K. minor* is reported from other Southern Ocean deep-sea holes for which diatom biostratigraphy is available, including ODP Holes 748B and 749A^63^. The best constrained Southern Ocean record of this species is from Hole 748B, where *K. minor* occurs within a narrow interval spanning Chron C10n. To refine this initial biomagnetostratigraphic framework, red–green–blue (RGB) data was extracted from the core images (Extended Data Fig. 2). We corrected the RGB data for overexposure in the centre of the images and underexposure at the edges of the images. The correction does not considerably change the main patterns in cyclicity. Please note that the main cyclicity in the core images is not an artefact of uneven lighting conditions but a true feature of the sediment cores. The resulting individual red, green, and blue records, were combined into a combined RGB record. High values in the combined RGB signal visually correspond to the lighter coloured strata, which at Site 699 are calcium carbonate dominated. At other South Atlantic and Pacific Ocean sites^3,10,64^, elevated levels of calcium carbonate (light-coloured sediments, RGB highs) correspond to eccentricity-paced productivity maxima that occur during an eccentricity minimum. The RGB and δ^18^O_calcite_ records both exhibit the dominant 110-kyr cyclicity but are not always perfectly aligned. To better visually align the high-amplitude mid-Oligocene ~110-kyr cycles identified at Sites 699, 1218, and 1264, we made a small correction to the Site 1264 eccentricity-tuned age model by changing the tie point ‘293.52 adjusted revised metres composite depth (armcd) − 27.511 Ma’ into ‘293.30 armcd − 27.511 Ma’. Both RGB and δ^18^O_calcite_ were considered when selecting our final tuning tie points (Supplementary Table 1). This tuning approach was independently validated by (1) the convincing alignment (within error) between the magnetostratigraphic reversals from Site 699A with those of the Westerhold et al.^3^ astronomically calibrated time scales (Extended Data Fig. 3), (2) the identification of between five and six precession forced cycles in the RGB record for some of the best-expressed ~110-kyr cycle (that is, the combined 95- and 125-kyr components of eccentricity) and (3) the coherency in the benthic foraminiferal δ^18^O stratigraphy from Site 699A and independently astronomically age-calibrated δ^18^O_calcite_ records from Walvis Ridge Site 1264, and equatorial Pacific Ocean Site 1218 (on the revised age model of Westerhold et al.^3^; Fig. 1).

Our fine-tuned astronomically calibrated age model for cores 699A-20H and 21H spans the 26.8- to 28.2-Ma interval and is based on 11 eccentricity based (La2011_ecc3L solution^57^) tie points and three magnetostratigraphic tie point (Extended Data Fig. 4 and Supplementary Table 1).

We note that the results of this study are independent from the adopted tuning approach as we used δ^18^O_calcite_ from Site 699 and Westerhold et al.^3^ to guide our sampling strategy and obtain temperature data for the targeted δ^18^O_calcite_ cycles.

### Sample preparation

The samples from cores 699A-20H and 21H are clay-rich and unlithified with generally well-preserved benthic foraminifera (Extended Data Figs. 5–7). Benthic foraminiferal abundance fluctuates substantially through the studied interval, with several intervals characterized by very low abundances. Levels of low foraminiferal abundance are at 182.6–182.8 and 192.1–192.6 meters below sea floor (mbsf). Benthic foraminiferal abundance can be lower owing to an increase in sedimentation rates, a drop in paleoenvironmental oxygen conditions or carbonate dissolution^65,66^. Based only on benthic foraminifera abundance variability in the samples, some degree of dissolution at discrete levels in the record cannot be ruled out. However, the generally good foraminiferal preservation through the record does not suggest major dissolution, as supported by scanning electron microscope images of specimens (Extended Data Figs. 5–7) taken from light (Extended Data Figs. 5 and 6) and dark (Extended Data Fig. 7) sediment intervals (Extended Data Fig. 2).

The samples were washed over a 63-μm mesh-size sieve with tap water and oven-dried at 40 °C. A total of 99 samples were picked for clumped isotope analysis from 179.9 to 195.45 mbsf. Benthic foraminifera were picked from the size fraction >150 μm after dry sieving. Benthic foraminifera were grouped according to taxonomy and ecology depending on species and genus abundance as follows: (1) *Cibicidoides* spp. (composed of *C. mundulus*, *C. eocaenus*, *C. havanensis*, *C. grimsdalei*, *C. micrus*, *C. brady*, *C. lamontdoherty* and *C. dickersoni*); (2) *Oridorsalis umbonatus*; (3) Epifaunals (composed of *Laticarinina pauperata*, *Gyroidinoides gyrardanus*, *G. planulatus*, *G. depressus*, *Nuttallides umbonifera*, *Alabamina weddellensis*, *Epistominella exigua*, *Anomalinoides rubiginosus* and *A. spissiformis*); (4) Nodosarids (*Nodosaria* spp., *Lenticulina* spp., Lagenidae and Polymorphinids), Pleurostomellids (*Pleurostomella* spp.) and Stilostomellids (*Stilostomella* spp.); and (5) Infaunals (*Pullenia bulloides*, *P. quinqueloba*, *Melonis barleanuum*, *Nonion havanense*, *Nonionella* spp. and *Globocassidulina subglobosa*). For several samples, this distinction into different species groups was not possible owing to extremely low foraminifera abundance, requiring all found specimens to be combined for measurements (samples 699A-20H-2, 20–22 cm to 699A-20H-2, 42–44 cm; samples 699A-20H-3, 5–7 cm to 699A-20H-3, 27–29 cm; samples 699A-21H-3, 10.5–13 cm to 699A-21-3. 50–53 cm). Foraminifera were cracked between glass plates and sonicated in deionized water (3 × 10–20 s) and methanol (1 × 5 s). At the end of the cleaning procedure, the test fragments were rinsed until the solute was no longer cloudy and oven-dried at 50 °C. Individual measurements from multiple adjacent samples were combined to calculate average Δ_47_ values, covering the minimum and maximum depth ranges of 10 and 30 cm, respectively, except between 182.21–182.67 mbsf (46 cm) and 186.45–187.05 mbsf (60 cm) where several samples were barren of foraminifera.

### Clumped isotope analyses

The clumped isotope paleothermometer relies on the thermodynamic bounding of ^13^C and ^18^O isotopes in calcite molecules as a function of ambient temperature and is unaffected by the δ^18^O_sw_ (ref. ^23^). The influence of non-thermal controls on Δ_47_, such as pH and the biological partitioning of isotopes in calcite, does not appear resolvable^67–69^. The low natural abundance of ^13^C–^18^O bonds within carbonate ions demands large sample sizes to produce data with the precision required for palaeoclimate applications. Here, we used small (~85 μg) carbonate samples and obtained the necessary precision by averaging a mean of 33 Δ_47_ measurement values (minimum 20 to maximum 46) from neighbouring samples^29,70^. The Δ_47_ measurements were performed at the Farlab, University of Bergen, on two Thermo Scientific MAT 253 Plus isotope ratio mass spectrometer connected to Thermo Scientific Kiel IV carbonate preparation devices. The analytical method used here is extensively described in Modestou et al.^30^ and Leutert et al.^71^. We used three carbonate standards (ETH 1, 2, 3), which differ in bulk isotopic composition and ordering state to correct for Δ_47_ scale compression and to transfer results to the Intercarb-Carbon Dioxide Equilibrium Scale (I-CDES)^72^. An additional standard (International Atomic Energy Agency (IAEA)-C2^72^) was not used for corrections but instead used to check the fidelity of the correction procedure (0.639 ± 0.027‰ (1 s.d.); *n* = 850). All analytical sessions (~23 h each) included approximately equal numbers of carbonate standards and samples. External reproducibilities (1 s.d.) in Δ_47_ of the four carbonate standards after correction were between 0.027 and 0.028‰.

### Oxygen and carbon isotopes

Oxygen and carbon stable isotopes were obtained from the same groups/specimens as a co-product of Δ_47_ measurements. Carbonate δ^18^O and δ^13^C values are reported relative to the Vienna Pee Dee Belemnite scale (VPDB) and were corrected with the same carbonate standards (ETH 1–3), using the values reported by Bernasconi et al.^73^, including a scale correction. The δ^18^O and δ^13^C values of all the standards have external reproducibilities (1 s.d.) better than or equal to 0.07‰ (δ^18^O) and 0.03‰ (δ^13^C).

To complement the stable isotope record obtained as a co-product of Δ_47_ measurements, stable isotopes were additionally measured on several monospecific samples of *Cibicidoides* (*C. munduls*/*C. eocaenus*) for the depth interval 193.6–194.1 mbsf and 194.7–195.0 mbsf at 10-cm resolution on a Thermo Scientific MAT 253 isotope ratio mass spectrometer connected to a Thermo Scientific Kiel IV carbonate preparation device at Farlab, University of Bergen. Carrara Marble (in-house CM12) was used as a working standard, and the values are reported relative to VPDB, calibrated using National Bureau of Standards (NBS) standards 18 and 19. External reproducibility in CM12 was better than or equal to 0.02‰ (δ^13^C) and 0.05‰ (δ^18^O) (1 s.d.) over the analysis interval.

### Clumped isotope temperatures

Owing to the large number of ‘replicate’ measurements required, Δ_47_ values from multiple 10-cm spaced samples were averaged for each data point, using the δ^18^O_calcite_ record as a guide to avoid aliasing and to obtain temperature data for the targeted δ^18^O_calcite_ cycles. On average, each temperature group combines measurements from three samples (from minimum two to maximum five), and a total of 32 temperature groups were obtained with this approach, equivalent to one temperature data point every 28 kyr.

Sample standard errors were determined by selecting the higher value between the sample standard deviation and the external reproducibility from IAEA-C2 (0.027‰) and calculating standard errors of the means depending on the number of measurements from each sample. Average Δ_47_ values for each group were determined as the average of all sample averages in the temperature group weighted by the number of measurements from the respective samples, with errors being estimated through the propagation of the standard errors of the samples. For each temperature group, a mean age was assigned by also averaging the age of each sample in the temperature group by the number of measurements.

Temperatures were then estimated with the calibration of Meinicke et al.^25^ updated to the I-CDES scale^74^:

1 $${\Delta }_{47}=(0.0397\pm 0.0011)\times {10}^{6}/{T}^{2}+(0.1518\,\pm \,0.0128)(T\,\mathrm{in}\,K).$$

Errors in temperature estimates (for example, propagated analytical and calibration errors) were determined through Monte Carlo simulations after Meckler et al.^37^. The final temperature errors are reported as 68% and 95% confidence intervals (Fig. 1).

To test the impact of the choice of calibration equation, we also calculated temperatures with the calibrations of Anderson et al.^45^ and Daëron and Vermeesch^46,75^ (Supplementary Table 2), which include both biogenic and inorganic (for example, natural and laboratory precipitate) samples. Temperatures calculated with these equations are on average 1.5 °C (ref. ^45^) and 1.2 °C (ref. ^46^) colder, respectively, than those obtained with equation (1), and the δ^18^O_sw_ on average 0.35‰ and 0.28‰ more negative. For this study, we chose to report temperatures with equation (1) because the other two calibrations include very high temperature (>100 °C) samples that can bias the relationship within the ocean temperature range. Although the choice of calibration affects our absolute reconstructed temperature and δ^18^O_sw_ (albeit within error of our estimates), the relative changes, which are the main focus of this study, are unaffected. We refrain from using the calibration of Daëron and Gray^76^ as it yields unrealistically cold temperatures when applied to the Plio-Pleistocene section of the Cenozoic record of Meckler et al.^37^.

In addition to the grouping (binning) approach to reconstruct temperature, we used a Gaussian moving window filter to independently confirm the validity of our temperature grouping. This verification process was carried out through a Monte Carlo simulation following the methodology outlined in Rodríguez-Sanz et al.^70^. In short, for each replicate analysis, we generated 10,000 random values on the basis of the observed external reproducibility of 0.027‰ from IAEA-C2 (Extended Data Fig. 8a), and assuming a normal distribution. To account for both analytical and calibration uncertainties, we computed 10,000 temperature estimates for each replicate using a random slope–intercept pair from the clumped isotope calibration equation (outlined below). Subsequently, we applied a Gaussian filter with a 110,000-year window to each simulation and calculated average temperatures every 25,000 years. The final temperature values are presented as the median (50th percentile), along with the associated uncertainties represented by the 95% and 68% confidence limits (Extended Data Fig. 8b). The filter generated a pattern remarkably similar to the temperature groups, indicating that our chosen binning approach did not introduce bias into the temperature reconstruction (Extended Data Fig. 8). Despite the similarity between the temperature patterns obtained with the binning and Gaussian filter approaches, we prefer the binning approach, as it enables variable resolution and minimal smoothing in places where data density is higher (such as between 27.55 and 27.4 Ma).

### δ^18^O_calcite_ and δ^18^O_sw_

To calculate δ^18^O_sw_, we used δ^18^O_calcite_ from the Δ_47_ analysis of *Cibicidoides* spp. for most of the samples that compose our temperature groups (86 of 106 samples). However, we also had to rely on other taxa when *Cibicidoides* spp. were absent or not sufficiently abundant for Δ_47_ analysis. Whereas Δ_47_ is not measurably impacted by using different benthic foraminifera taxa^77^, there are species-specific offsets in δ^18^O_calcite_ (for example, refs. ^78,79^) that need to be corrected for. Our Site 699 dataset shows distinct offsets between the δ^18^O_calcite_ of *Cibicidoides* spp. and the other taxonomic groupings used as Δ_47_ aliquots from the same samples (Extended Data Fig. 9a). Averaged across all samples with dual measurements, aliquots of mixed epifaunal benthic foraminifera species were offset in δ^18^O_calcite_ by −0.13‰ (±0.05, 95% confidence intervals), *O. umbonatus* by −0.22‰ (±0.04, 95% confidence intervals) and mixed samples of all benthic foraminiferal species by −0.15‰ (±0.09, 95% confidence intervals) with respect to *Cibicidoides* spp. (Extended Data Fig. 9b). We used these mean offsets to correct δ^18^O_calcite_ with respect to *Cibicidoides* spp. when *Cibicidoides* spp. was not available and used the corrected δ^18^O_calcite_ value from the other species/groups with the following order of priority where multiple options were available: (1) mixed epifaunal benthic foraminifera (8/106 samples), (2) *O. umbonatus* (1/106) and (3) mixed benthic foraminifera, in case of very low abundance of specimens (11/106) (Extended Data Fig. 9c). We preferred the mixed epifaunal group over *O. umbonatus*, as the former consistently display the smallest offset from *Cibicidoides* spp. It is also worth noting that the offset correction ultimately has limited impact on the Site 699 δ^18^O_calcite_ record and, hence, δ^18^O_sw_, owing to the relatively small number of samples lacking *Cibicidoides* spp. and the small magnitude of the δ^18^O_calcite_ offsets that we corrected for, relative to the changes observed in the δ^18^O_calcite_ record (Extended Data Fig. 9d). This notion is corroborated by the similar magnitude of δ^18^O_calcite_ fluctuations at Site 699 and at Sites 1264 (South Atlantic) and 1218 (Equatorial Pacific) where *Cibicidoides mundulus* and *Cibicidoides* spp. were used, respectively, with a mean difference of only 0.18‰.

To obtain δ^18^O_sw_, we calculated the average δ^18^O_calcite_ for each group using the average δ^18^O_calcite_ value for each sample in a temperature group and weighing these in the identical way to the Δ_47_ data (that is, giving more weight to samples that yielded more measurements for Δ_47_). This way, any skewing of the group averages towards a given sample owing to an unequal number of Δ_47_ measurements is also reflected in the δ^18^O_calcite_ and, as a result, δ^18^O_sw_.

The temperature and the average δ^18^O_calcite_ for each group were combined to obtain δ^18^O_sw_ following the equation of Marchitto et al.^20^

2 $$({{\rm{\delta }}}^{18}{{\rm{O}}}_{\mathrm{calcite}}-{{\rm{\delta }}}^{18}{{\rm{O}}}_{\mathrm{sw}}+0.27)=-0.245\times \mathrm{BWT}+0.0011\times {\mathrm{BWT}}^{2}+3.58,$$

where BWT is bottom water temperature.

### Statistical analysis

We tested the statistical significance of the temperature and δ^18^O_sw_ changes associated with the six 110-kyr eccentricity cycles between 28.2 and 27.2 Ma using mean values of temperature and δ^18^O_sw_ groups (Extended Data Fig. 10). The temperatures and δ^18^O_sw_ coinciding with the cycle core ~40 kyr of peak eccentricity values were grouped in the ‘eccentricity maxima’ group of values and tested against background values (Extended Data Fig. 10). In doing so, we took into account a slight (±5 kyr) misalignment between the data and the eccentricity cycles. The test was performed with a *t*-test using the R stats package R Core Team^80^ (Extended Data Fig. 10b,d). For temperature, the *t*-test returned a *P* of 0.011, meaning the mean of the groups are statistically different. For δ^18^O_sw_, the *t*-test returned a *P* value of 0.256, meaning the mean of the groups are not statistically different (Extended Data Fig. 10b,d).

## Online content

Any methods, additional references, Nature Portfolio reporting summaries, source data, extended data, supplementary information, acknowledgements, peer review information; details of author contributions and competing interests; and statements of data and code availability are available at 10.1038/s41561-025-01878-y.

## Supplementary information

Supplementary Information Supplementary Table 1.

Supplementary Data 1 Raw dataset and all data presented and discussed in the text and Methods.

## Data Availability

All data are available in Supplementary Table 2 and in the EarthChem data repository at 10.60520/IEDA/114211 (accessed 31 October 2025).
