# Supplementary material for: Oligocene deep ocean oxygen isotope variations primarily driven by temperature
Source: Nat Geosci. 2026 Jan 7;19(2):209–15. doi: 10.1038/s41561-025-01878-y (PMC12893918; doi:10.1038/s41561-025-01878-y)
Supplement: Supplementary file 1 — Supplementary Table 1. [file 41561_2025_1878_MOESM1_ESM.pdf]

nature geoscience

Supplementary information https://doi.org/10.1038/s41561-025-01878-y

Oligocene deep ocean oxygen isotope variations primarily driven by temperature

In the format provided by the authors and unedited

**Depth (meters)**	**Tuned age (Million years)**	**Tie Point Comment**
166.950	26.599	PMAG (Paleomagnetic stratigraphy)
172.513	26.805	RGB tuned, $\delta^{18}O$ and PMAG guided
174.301	26.912	RGB tuned, $\delta^{18}O$ and PMAG guided
175.840	27.020	RGB tuned, $\delta^{18}O$ and PMAG guided
179.900	27.192	RGB tuned, $\delta^{18}O$ and PMAG guided
181.542	27.298	RGB and $\delta^{18}O$ tuned, PMAG guided
182.993	27.407	RGB and $\delta^{18}O$ tuned, PMAG guided
185.100	27.511	RGB tuned, $\delta^{18}O$ and PMAG guided
186.473	27.590	RGB tuned, $\delta^{18}O$ and PMAG guided
189.340	27.847	PMAG
190.296	27.897	RGB tuned, $\delta^{18}O$ and PMAG guided
193.984	28.058	RGB tuned, $\delta^{18}O$ and PMAG guided
196.286	28.167	RGB tuned, $\delta^{18}O$ and PMAG guided
198.350	28.263	PMAG

Supplementary Table 1. Age model tie points.
